# Genetic Diversity, Population Structure, and Linkage Disequilibrium of an Association-Mapping Panel Revealed by Genome-Wide SNP Markers in Sesame

**DOI:** 10.3389/fpls.2017.01189

**Published:** 2017-07-06

**Authors:** Chengqi Cui, Hongxian Mei, Yanyang Liu, Haiyang Zhang, Yongzhan Zheng

**Affiliations:** ^1^National Key Laboratory of Crop Genetics and Germplasm Enhancement, Cotton Research Institute, Nanjing Agricultural UniversityNanjing, China; ^2^Henan Sesame Research Center, Henan Academy of Agricultural SciencesZhengzhou, China; ^3^Key Laboratory of Oil Crops in Huanghuaihai Plain, Ministry of AgricultureZhengzhou, China; ^4^Henan Provincial Key Laboratory for Oil Crops ImprovementZhengzhou, China

**Keywords:** *Sesamum indicum* L., SNPs, genetic diversity, population structure, linkage disequilibrium

## Abstract

The characterization of genetic diversity and population structure can be used in tandem to detect reliable phenotype–genotype associations. In the present study, we genotyped a set of 366 sesame germplasm accessions by using 89,924 single-nucleotide polymorphisms (SNPs). The number of SNPs on each chromosome was consistent with the physical length of the respective chromosome, and the average marker density was approximately 2.67 kb/SNP. The genetic diversity analysis showed that the average nucleotide diversity of the panel was 1.1 × 10^-3^, with averages of 1.0 × 10^-4^, 2.7 × 10^-4^, and 3.6 × 10^-4^ obtained, respectively for three identified subgroups of the panel: Pop 1, Pop 2, and the Mixed. The genetic structure analysis revealed that these sesame germplasm accessions were structured primarily along the basis of their geographic collection, and that an extensive admixture occurred in the panel. The genome-wide linkage disequilibrium (LD) analysis showed that an average LD extended up to ∼99 kb. The genetic diversity and population structure revealed in this study should provide guidance to the future design of association studies and the systematic utilization of the genetic variation characterizing the sesame panel.

## Introduction

Sesame (*Sesamum indicum* L., 2n = 26), a member of the Pedaliaceae family, is one of the most ancient oil crops and it is grown widely in both tropical and subtropical areas (Bedigian and Harlan, 1986; Ashri, 1998). Sesame seed is now widely used in food, nutraceutical, pharmaceutical, and industry in many countries. Compared with peanut (*Arachis hypogaea*), soybean (*Glycine max*), oilseed rape (*Brassica napus*), sunflower (*Helianthus annuus* L.) and other oilseed crops, sesame seeds innately contain a higher oil content (∼55% of dry seed), in which the oleic acid (18:1) (32∼46%) and linoleic acid (18:2) (42∼59%) components follow a near 1:1 ratio (Uzun et al., 2008). In addition, sesame oil contains rich antioxidant lignans, such as sesamin and sesamolin, which are known to play important roles in food processing and human healthcare (Namiki, 1995; Anilakumar et al., 2010).

The cultivation history of sesame dates back to between 5,000 and 5,500 years ago in the Harappa Valley of the Indian subcontinent (Bedigian and Harlan, 1986). Under long-term natural and artificial selection, coupled to a wide geographic distribution, many diverse variations have accumulated in sesame, which could serve as important resources for genetic investigations and crop breeding objectives (Wei et al., 2015). In the past 19 years, great effort has been devoted to collecting and preserving sesame in India, China, and Korea (Zhang H. et al., 2012). Bisht et al. (1998) reported that 6,658 accessions were conserved in the Indian collection alone. In South Korea, the number of germplasm accessions has reached 7,698 (Park et al., 2015). In China, two sets of sesame germplasm collections are preserved by the Oil Crops Research Institute, Chinese Academy of Agricultural Sciences (OCRI-CAAS), and by the Henan Sesame Research Center, Henan Academy of Agricultural Sciences (HSRC-HAAS). This OCRI-CAAS collection contains 4,251 accessions, most of which were sent to the National Genebank for long-term storage, and a core collection of 453 representative samples that was established in 2000 (Zhang et al., 2000). With support from the China Agriculture Research System (CARS-15), there has been an extensive collecting effort conducted in the past 7 years: to date more than 5,200 accessions are now preserved in the HSRC-HAAS collection, and a core collection of 501 representative samples was amassed by Liu et al. (2017).

Knowledge of the genetic diversity and relationships among germplasm accessions is of vital importance for improving the sesame varieties. Molecular markers reflect the actual level of genetic variation existing among genotypes at the DNA level; hence, they provide a more accurate estimate of such variation than do either phenotypic or pedigree information (Pham et al., 2009). In sesame, the DNA markers such as amplified fragment length polymorphism (AFLP) (Laurentin and Karlovsky, 2006), sequence-related amplified polymorphisms (SRAP) (Zhang et al., 2010; Zhang H. et al., 2012), and inter-simple sequence repeat (ISSR) (Kumar et al., 2012) have been used for the analysis of germplasm genetic diversity and in cultivar fingerprinting. Recently, the rapid development and application of sequence-specific markers such as genomic simple sequence repeats (SSR; Cho et al., 2011), expressed sequence tag (EST)-SSR (Wei et al., 2008; Zhang Y. et al., 2012), insertions and deletions (InDels) (Wu et al., 2014) were also reported for sesame. However, a limited number of selected markers used in these studies might provide biased estimates of genetic variability (Queirós et al., 2015).

Single-nucleotide polymorphisms (SNPs) based on next generation sequencing (NGS) are more useful than conventional markers. This is because SNPs are extremely abundant, and the number of unbiased SNPs can nowadays easily been obtained for non-model species (Fischer et al., 2017). With the advent of NGS technologies and a substantial reduction in the costs of sequencing, SNPs are being discovered and genotyped in a high-throughput way (Davey et al., 2011). The specific-locus amplified fragment sequencing (SLAF-seq) method, which combines NGS with restriction enzyme digestions to reduce the complexity of the target genomes, has several positive characteristics: namely, a low cost, high efficiency, and enhanced accuracy in genome-wide marker development and genotyping (Sun et al., 2013). Not surprisingly, the SLAF-seq approach is increasingly being used in high-density genetic map constructions and genome-wide association studies (GWAS; Qi et al., 2014; Zhao et al., 2015; Zhen et al., 2016; Mei et al., 2017).

In this study, 366 elite germplasm accessions were selected from the HSRC-HAAS primary core collection and genotyped using the SLAF-seq method to evaluate their usability as an association-mapping panel. Our objectives were as follows: (a) to assess the genetic diversity as represented by the 366 sesame germplasm accessions; (b) to calculate the characteristics of the population structure; and (c) to estimate the linkage disequilibrium (LD) patterns occurring in the sample of sesame germplasm.

## Materials and Methods

### Sesame Plant Material

To assemble the association-mapping panel consisting of 366 germplasm accessions (taken from the HSRC-HAAS collection), we used 329 accessions coming from 18 provinces in China, plus another 37 accessions that were introduced from 11 countries (Supplementary Table 1). All of these accessions are able to flower and ripen under natural conditions in temperate regions, which would allow us to precisely evaluate their traits in field trials. Their wide geographic distribution and phenotype variation makes these germplasms an ideal model for exploring the genetic diversity of sesame.

### DNA Extractions and SLAF-seq

Genomic DNA was extracted from the young leaves of each accession by using the CTAB method (Paterson et al., 1999) with slight modifications to the CTAB buffer components to eliminate any ultra-plentiful polysaccharides in the sesame leaves (Mei et al., 2017). Crude DNA samples were purified using a DNeasy Kit (Qiagen, Valencia, United States), then assessed by electrophoresis on 0.8% agarose gel and quantified using spectrophotometry (NanoDrop 8000, Thermo Scientific, United States). The SLAF libraries were constructed following the procedure described by Sun et al. (2013), except that here two restriction enzymes, *Hae*III [recognition site 5′-GG/CC-3′, New England Biolabs (NEB), United States) and *Hpy*166II (5′-GTN/NAC-3′, NEB), were used to digest the genomic DNA. Pooled samples were separated by 2% agarose gel electrophoresis, and those fragments ranging from 264 to 364 base pairs (with indexes and adaptors) in size were excised and purified using a QIAquick gel extraction kit (Qiagen, Hilden, Germany). The gel-purified products were diluted and subjected to pair-end sequencing on an Illumina HiSeq 2000 platform (Illumina Inc., San Diego, United States) at Beijing Biomarker Technologies Corporation^1^.

### SLAF-tag Grouping and SNP Calling

The high-quality paired-end reads were aligned with an improved Zhongzi No. 13 genome assembly (Cui et al., submitted) coupled to Burrows–Wheeler Aligner (BWA) software (Li and Durbin, 2009) that was set to the default parameters. The SLAF-tag groups were generated by reads that were mapped onto the same position. The SNP detection was performed using a Genome Analysis Toolkit (GATK, McKenna et al., 2010) and SAMtools (Li, 2011). The process went as follows (Zhou et al., 2015): (1) after the BWA alignment, the reads around the InDels were realigned by the GATK; (2) the SNPs were called at a population level with the GATK and SAMtools. For GATK, the SNP confidence score was set as >30, and the parameter “-stand_call_conf” to a value of 30. The same realigned BAM files were used in the SNP calls with the SAMtools “mpileup” package; (3) in the filter step, the common sites identified by the GATK and SAMtools were chosen using the SelectVariants package.

### Data Analysis

To reduce the influence of a strong LD on the assessment of population stratification, a subset of 12,178 SNPs were selected by setting an LD (*r*^2^) threshold to 0.1 for the SNP pairs in a sliding window of 50 SNPs—by using PLINK v.1.07 (Purcell et al., 2007). The parameter was: “plink –file data –indep-pairwise 50 10 0.1.” Population structure was estimated with 12,178 SNPs by using a Bayesian model-based program implemented in STRUCTURE 2.3.4 (Pritchard et al., 2000). Three runs were performed for each number of populations (*K*) set from 1 to 10. The burn-in time and the Bayesian Markov Chain Monte Carlo (MCMC) replication number were both set to 100,000 for each run. The most likely *K* value was determined by the log likelihood of the data [LnP(D)] and an *ad hoc* statistic, Δ*K*, in the program Structure Harvester (Evanno et al., 2005). Lines with a probability of membership ≥70% were assigned to a subgroup, whereas those with <70% were assigned to a “Mixed” subgroup (Liu et al., 2003).

A principal components analysis (PCA) of the selected SNPs was performed with the EIGENSOFT software (Price et al., 2006). To construct the neighbor-joining tree, the PHYLIP software was used (Plotree and Plotgram, 1989). An analysis of molecular variance (AMOVA) and the population pair-wise *F* statistics (*F*_ST_) were calculated by using the software Arlequin v.3.52 (Excoffier and Lischer, 2010). According to the standard described by Del Carpio et al. (2011), there is no differentiation between the subpopulations when *F*_ST_ = 0, but complete differentiation occurs between the subpopulations when *F*_ST_ = 1. Populations were considered to have little differentiation when *F*_ST_ ≤ 0.05, moderate differentiation when 0.05 < *F*_ST_ ≤ 0.15, strong differentiation when 0.15 < *F*_ST_ ≤ 0.25, and very strong differentiation when *F*_ST_ > 0.25 (Hartl, 1980; Mohammadi and Prasanna, 2003).

Genome-wide LD was estimated in the total panel and for each subgroup (as determined by the population structure) by pairwise comparisons among the 44,109 SNP markers [missing rates <0.30 and minor allele frequency (MAF) ≥0.05] using *r*^2^. For all pairs of SNPs, the *r*^2^ was calculated using the PopLDdecay v.3.26 program^2^.

## Results

### SLAF-Seq Genotyping

A total of 81.2 Gb of sequence data, including 902.36 million pair-end reads, were generated by the SLAF-seq when applied to the 366 sesame germplasm accessions. The Q30 ratio and guanine-cytosine (GC) content were 84.82% and 39.47%, respectively (Supplementary Table 2). High quality reads were aligned to the improved Zhongzi No. 13 genome assembly, and a total of 202,603 SLAFs evenly distributed across the whole genome were identified. SLAFs that were used for calling the SNPs had an average depth of 15.32-folds per individual. A total of 722,824 SNPs were initially called for these accessions. After removing those nucleotide polymorphisms that had missing rates >0.30, a set of 138,029 SNPs was generated. The allele frequency for each SNP site was then calculated: the MAF of the SNPs varied from 0.30 to 49.86%, with an average of 7.76%, and ∼68.04% of the SNPs had a low frequency (MAF < 0.05) across the 366 accessions (Supplementary Figure S1). After excluding the SNPs with a MAF < 0.01, there were left 89,924 (∼65.15%) high-quality SNPs (Supplementary Table 3) evenly distributed across the whole genome that could be used for further analysis.

**FIGURE 1:**
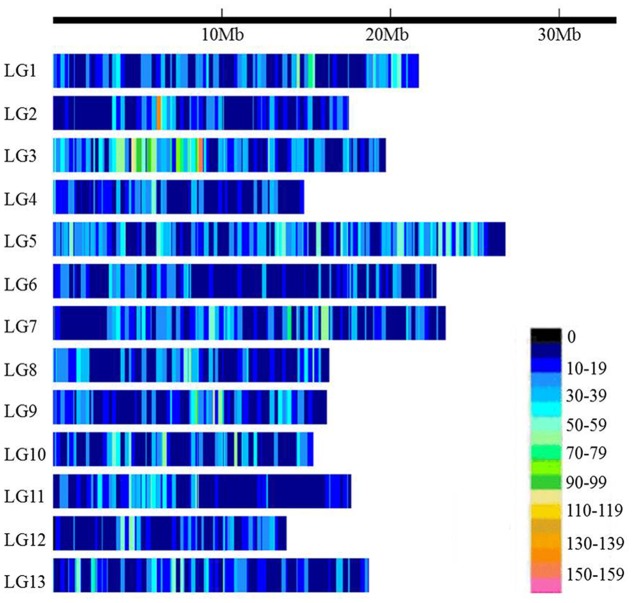
Single-nucleotide polymorphism (SNP) distributions on the 13 linkage groups (LGs) of sesame. The horizontal axis shows the LG length; the 0∼159 legend insert depicts the SNP density (the number of SNPs per 50 kb window).

The 89,924 high-quality SNPs covered all 13 linkage groups (LGs) (**Figure 1** and **Table 1**). The largest number of SNPs was found on LG5 (12,491 SNPs) followed by LG3 (11,351 SNPs), whereas the smallest number of SNPs occurred on LG4 (4,028 SNPs). The number of SNPs on each chromosome was consistent with the physical length of the respective chromosome. The average marker density was approximately 2.67 kb/SNP. LG6 had the lowest SNP marker density (3.96 kb/SNP), and LG3 had the highest marker density (1.70 kb/SNP).

**Table 1:** Summary of the number of SNPs mapped onto each linkage group (LG) and the gene diversity and LD decay estimated for each LG.

LG	Number of SNPs	Density of SNP (kb/SNP)	Gene diversity	LD decay (kb)
LG1	8,416	2.53	0.13	75
LG2	6,193	2.76	0.14	97
LG3	11,351	1.70	0.18	84
LG4	4,028	3.59	0.20	104
LG5	12,491	2.11	0.16	66
LG6	5,637	3.96	0.15	255
LG7	9,202	2.49	0.17	107
LG8	5,180	3.09	0.18	105
LG9	5,788	2.74	0.25	85
LG10	6,244	2.41	0.13	82
LG11	5,451	3.18	0.18	67
LG12	4,355	3.10	0.17	82
LG13	5,606	3.29	0.18	106
Total	89,924	2.67	0.17	99

### Population Structure of the Association-Mapping Panel

**FIGURE 2:**
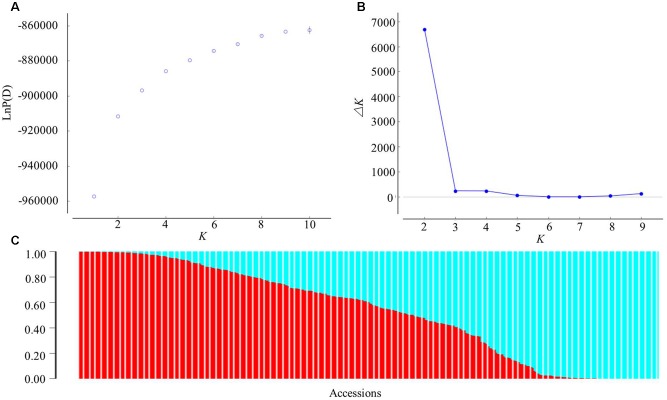
Analysis of the population structure of the 366 sesame accessions using STRUCTURE. **(A)** Estimated LnP(D) of possible clusters (*K*) from 1 to 10; **(B)** Δ*K* based on the rate of change of LnP(D) between successive *K*; **(C)** population structure based on *K* = 2. In panel **(C)**, each individual is represented by a vertical bar partitioned into two colored segments, with their respective lengths representing the proportion of the individual’s genome in a given group: red, for Pop 1; blue, for Pop 2.

A subset of 12,178 SNPs was selected for analysis of the population structure. The hierarchical population structure was determined for the entire panel via the model-based STRUCTURE program, but without providing any information per se on the population structure. As *K* changed from 1 to 10, the log likelihood value [LnP(D)] increased continuously and an inflection was evident when *K* increased numerically from 1 to 2 (**Figure 2A**). Thus, the most likely numerical value of *K* was 2. The number of subgroups (*K*) was further validated by the second-order statistics of Δ*K*. The Δ*K* value showed a peak at *K* = 2 (**Figure 2B**), which supported the classification of the panel into two major subgroups (**Figure 2C**).

When using a probability of membership threshold of 70%, 144 and 111 accessions were respectively assigned into the two subgroups, Pop 1 and Pop 2, while the remaining 111 accessions were classified into a mixed subgroup (Mixed) (Supplementary Table 1). Most accessions of Pop 1 came from the Southern areas in China, while 14 accessions in total came from Bangladesh (*n* = 2), Japan (1), India (2), Burma (2), Thailand (1), Arab Emirates (2), Mexico (1), Mozambique (2), and Guinea (1). The remainder of Pop 1 comprised 12 provinces in China (eight Southern provinces and four Northern provinces). The accessions of Pop 2 were mainly collected from the Northern areas in China; specifically, just three accessions came from South Korea, Japan, and United States, whereas 108 accessions came from nine provinces in China (eight Northern provinces and one Southern province). For the Mixed group, its accessions were collected from both the Southern (23 from five provinces) and Northern areas (68 from five provinces) in China, along with 20 accessions coming from Japan (12), Burma (3), Mozambique (1), Arab Emirates (1), United States (2), and Thailand (1).

**FIGURE 3:**
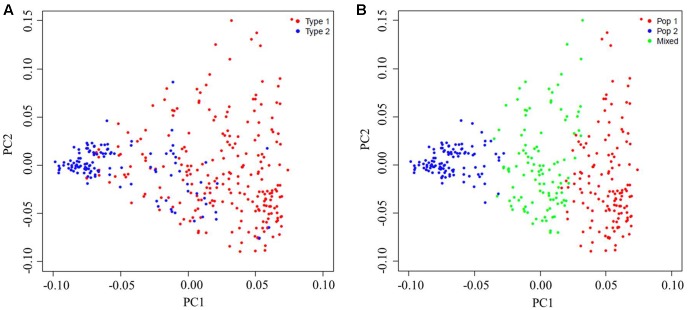
Principal components analysis (PCA) of the population for 366 sesame accessions based on 12,178 single-nucleotide polymorphisms (SNPs). Each individual is represented by one dot, with its symbol color corresponding to the assigned subgroup classification. **(A)** PCA plots of the sesame germplasm collection based upon their geographic origins: Type 1, the Southern-area subgroup, and Type 2, the Northern-area subgroup. **(B)** PCA plots of the same sesame germplasm collection but now based on subgroups as identified by STRUCTURE.

The PCA was done to further assess the population subdivisions. PC1 explained 10.10% of the genetic variation found, while PC2 and PC3 explained 6.77 and 4.40% of the variation, respectively. There was a significant correlation between PC1 and distributional latitudes of the accessions (Pearson’s correlation test, *r*^2^ = 0.37, *P* < 0.0001). Therefore, the sesame panel along the PC1 axis could be separated into Type 1 (a Southern-area subgroup) and Type 2 (a Northern-area subgroup) subpopulations (**Figure 3A** and Supplementary Table 1), which corresponded to those accessions collected from the Southern and Northern areas in China, respectively. However, some intermediate lines made the grouping less than clear-cut. When considering these intermediate lines, the panel could be neatly divided into three clusters (**Figure 3B**) corresponding to the three subgroups (**Figure 2C**) as inferred by using STRUCTURE.

**FIGURE 4:**
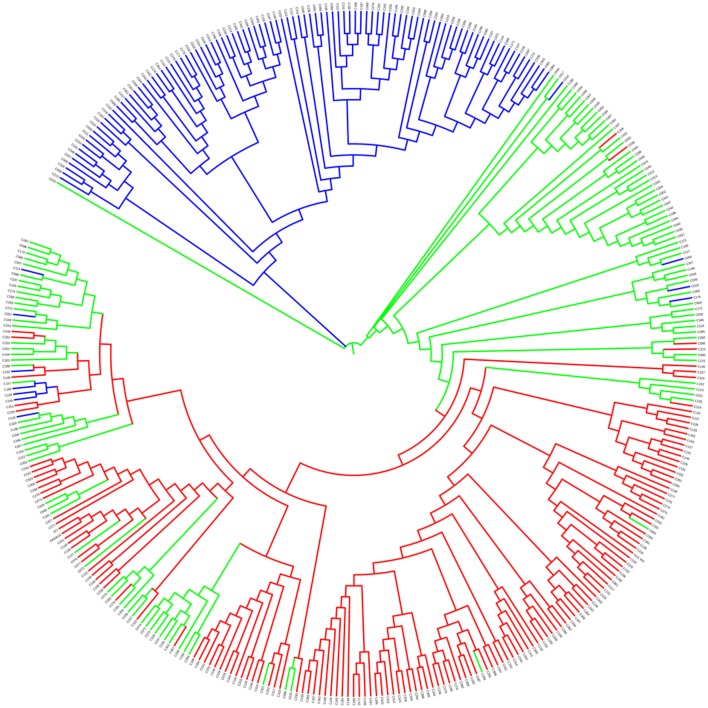
Neighbor-joining phylogenetic tree of the 366 sesame accessions. Red indicates Pop 1, blue indicates Pop 2, and green indicates the Mixed group.

The neighbor-joining phylogenetic tree was built to search for genetic relationships among the sesame accessions in the panel. This panel was grouped into two recognizable clusters—blue vs. green/red clusters shown in **Figure 4**—of which the blue cluster tended to be from the Northern areas of China. Similarly, the accessions from the Southern areas tended to be clustered together (red, **Figure 4**) while those accessions belonging to the Mixed group were distributed across the whole phylogenetic tree (green, **Figure 4**).

The genome-wide data set of 89,924 SNP markers with an MAF ≥ 0.01 was used to estimate the genetic differentiation for the panel. The AMOVA results indicated that 14.92% of the total genetic variation occurred among the subgroups. While a larger amount of variation (63.06%) was among individuals within the populations, 22.03% of variation was found within the individuals. The measure of population differentiation, *F*_ST_, among the subgroups was 0.15 highly significant at *P* < 0.0001. The corresponding *F*_ST_ values between the subgroups ranged from moderate, for Pop 1 vs. Mixed (0.07, *P* < 0.0001) and Pop 2 vs. Mixed (0.11, *P* < 0.0001), to very strong (0.26, *P* < 0.0001) for that of Pop 1 vs. Pop 2.

### Genetic Diversity Revealed by SNP Markers

A total of 89,924 SNPs were used to study the genetic diversity of the sesame panel. The average gene diversity of the panel was 0.17; the highest was 0.25 on LG9, followed by LG4 (0.20), with the lowest value found (0.13) on LG1 and LG10 (**Table 1**). The ranges for the estimates of gene diversity in Pop1, Pop2, and the Mixed were 0–0.66, 0–0.66, and 0–0.67, respectively, and their corresponding averages were 0.17, 0.11, and 0.17. On the basis of these SNP data, the sequence diversity (π) was estimated as 1.1 × 10^-3^ for all of the sesame accessions, and as 1.0 × 10^-4^, 2.7 × 10^-4^, and 3.6 × 10^-4^ for Pop 1, Pop 2, and the Mixed group, respectively.

To reveal the genetic differences among the different groups of the sesame germplasm, a comparative analysis of their allele frequencies was performed. The Pop 1 accessions had the largest number of group-specific SNPs (*n* = 3,720 SNPs), followed by Pop 2 (290), while the Mixed accessions had the smallest number of group-specific SNPs (157). In the pairwise comparisons of Pop 1, Pop 2, and the Mixed group, the number of SNPs unique to the Pop 1 (31,203) vastly exceeded those unique to the Pop 2 (2,195), though the number of group-specific SNPs (4,833) in Pop1 was just a little more than twice that in the Mixed group (2,352), whereas the number of SNPs unique to the Mixed group (27,640) was far greater than those unique to the Pop 2 (1,403).

The low level of heterozygous genotypes (heterozygosity = 0.05) was consistent with the inbreeding nature of sesame. The average heterozygosity was higher in Pop 2 (= 0.05) than in Pop 1 (= 0.04). The genetic distance among the 366 sesame accessions averaged 0.17, with a range of 0.01–0.42. Nonetheless, Pop 2, which included accessions collected from the Northern areas in China, had the lowest average genetic distance, at 0.11, as well as the smallest range, from 0.01 (C020 and C022) to 0.16 (C027 and C125). By contrast, Pop 1 and the Mixed group had a similar average genetic distance (at 0.17 and 0.16, respectively), which ranged from 0.01 (C150 and C255) to 0.40 (C278 and 377), and from 0.05 (C245 and C247) to 0.30 (C076 and C181), respectively.

### Linkage Disequilibrium across Whole Sesame Genome

**FIGURE 5:**
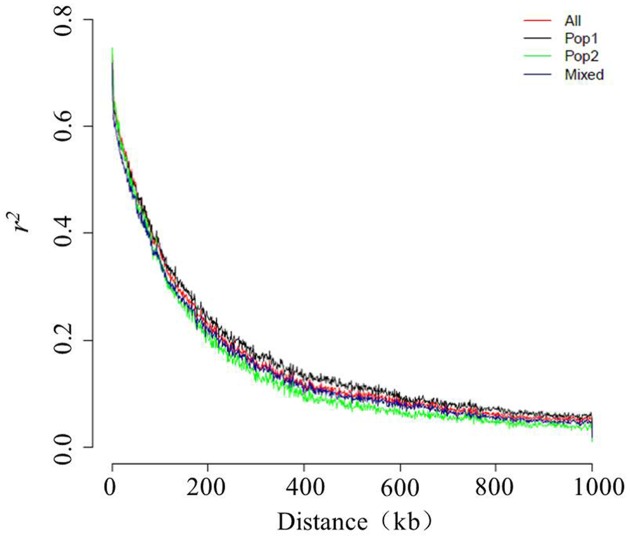
Genome-wide decay of the linkage disequilibrium (LD) in the sesame genome.

In this panel, the genome-wide LD decay at which *r*^2^ decreased to 0.37 from the initial value of 0.74 was ∼99 kb, such that the *r*^2^ dropped to 0.2 at ∼234 kb and to 0.1 at ∼467 kb (**Figure 5**). To obtain more details of this LD behavior, the chromosome-wise LD between the SNP pairs was also calculated. The highest maximum average LD (*r*^2^ < 0.80) was found for LG1, and the lowest maximum average LD (*r*^2^ = 0.66) was found for LG7. Except for the latter, all of the other LGs showed a maximum average LD that exceeded 0.70. The highest LD decay was ∼255 kb for LG6, followed by that seen for LG7 (∼107 kb), whereas the lowest LD decay (∼66 kb) was observed for LG5 (Figure S2). When the population-wise LD was calculated, the maximum average LD (*r*^2^ = 0.75) occurred in Pop 2, for which the LD decay was ∼83 kb. However, the minimum average LD was found to be very low (0.04) for Pop 2. The maximum average LD for the Mixed group was comparatively lower (0.72), with the decay to half to its initial value occurring at ∼86 kb. The average minimum LD was also very low (0.04) for the Mixed subgroup. The maximum and minimum average LD values for Pop 1 were 0.74 and 0.06, respectively. Compared with the LD decays of the two other subgroups, Pop 1 had the highest rate of LD decay, at ∼100 kb (**Figure 5**).

## Discussion

### Genetic Diversity Revealed by SNP Markers

The genetic diversity housed in germplasm collections is an important foundation for crop improvement and a key component of plant conservation and breeding strategies (Thomson et al., 2007). For sesame, its genetic diversity has been assessed in several previous studies. For example, Zhang et al. (2010) reported an average gene diversity of 0.24 in 404 accessions, as based on SRAPs and SSRs, while Cho et al. (2011) found a lower gene diversity, of 0.17, in a sesame panel based solely on SSRs. In general, the level of genetic diversity estimated by a limited number of SNPs tends to be lower than that estimated through the use of SSR markers (Jones et al., 2007; Van Inghelandt et al., 2010). However, in considering the criteria for genetic diversity, more weight should have been given to the number of loci instead of the number of alleles (Lu et al., 2009). So long as a sufficiently large number of unbiased NGS-based SNPs are analyzed across the genome, the SNP estimates will accurately reflect the genome-wide diversity occurring in natural populations (Fischer et al., 2017).

In our study, the 89,924 SNPs identified by SLAF-seq were evenly distributed across the whole genome of sesame. Compared to the SSR markers used in prior studies, these SNPs derived via SLAF-seq apparently could provide a better coverage of the genome and unbiased estimates of its diversity. By using the number of SNPs, the average gene diversity of 0.17 was calculated for the panel. In the present study, the average nucleotide diversity was 1.1 × 10^-3^, a value similar to that reported by both Wei et al. (2015) and Wang et al. (2014); this indicates that the present panel had a modest level of nucleotide diversity and so it could be considered as a representative sample to conduct GWAS. However, as cultivars derived from a single taxon (Bedigian and Harlan, 1986; Bedigian, 2010), the average nucleotide diversity (1.1 × 10^-3^) of sesame was lower than that of rice (Huang et al., 2010), yet similar to that of the soybean landraces which are considered to originate from a single domestication event (Zhou et al., 2015).

The proportion of rare SNPs (i.e., MAF < 0.05) we examined amounted to ∼68.04%, which was similar to those reported for the genomes of Arabidopsis and Alfalfa (Clark et al., 2007; Zhang et al., 2015). The high proportion of rare SNPs in our study may have two explanations. Firstly, since the SNPs were identified via SLAFs evenly distributed across the whole genome, they should be less prone to bias than would be low-coverage sequencing data (Huang et al., 2012). Secondly, in following its recent program to conserve genetic resources, a significant number of minor sesame varieties have been collected and preserved by HSRC-HAAS. For the LD-based mapping, markers with rare alleles are thus more likely to result in spurious findings. The SNPs with a MAF < 0.05 were removed in several previous studies (Huang et al., 2010; Yano et al., 2016). However, rare SNPs may also have an effect on the expression of a specific phenotype (Song et al., 2015). Given that the number of individuals with a specific genotype can be very small, the effect of rare alleles on genome mapping could extend beyond the effect of just small population sizes. In such cases, increasing the number of individuals with rare alleles could improve the power to test these rare alleles. Fortunately, a biparental population-based mapping approach, especially as done through the Nested Association Mapping (NAM), is able to use alleles occurring at a low frequency in natural populations by designing crosses to create artificial populations that have inflated frequencies of those alleles (Lu et al., 2010; Tian et al., 2011).

Sesame is a mostly self-pollinated plant, and hence it is likely that all of the sesame accessions in the present study had been held for many generations via self-pollination. Their genomes are thus expected to be mostly homozygous. In line with this expectation, the average heterozygosity in the sesame panel was 0.046; this suggests that the accessions we used were quite close to being inbred lines. Hence, the accessions selected from the HSRC-HAAS core collection are useful and suitable for investigating multiple phenotypic traits in a multi-plot field test over several years and to also carry out GWAS.

### Population Structure of the Association-Mapping Panel

The complex breeding history of many important crops and the limited gene flow in most wild plant populations have created complex structures within their germplasms (Sharbel et al., 2000). Detailed knowledge about the population structure in an association panel is thus important to avoid any spurious associations (Flint-Garcia et al., 2005). An assessment of structure in sesame has been reported by using different populations. For instance, Ali et al. (2007) found that 96 sesame accessions, collected from different parts of the world, could be separated into just two major groups that discriminated varieties as related to their geographical origin. Recently, Wei et al. (2015) divided 705 sesame accessions into two clusters by using a neighbor-joining tree. Similarly, in our study, the *K* value of 2 was determined by both the LnP(D) and Δ*K*. By using a 70% probability of membership threshold, the panel was successfully divided into three subgroups (Pop 1, Pop 2, and the Mixed). Most of the accessions belonging to Pop 1 came from Southern areas whereas the accessions of Pop 2 came from Northern areas in China. The remaining 111 accessions were all collected from both Southern and Northern areas, thus showing the substantial exchange of germplasm that has occurred in sesame.

The PCA was performed to examine the population structure of the sesame panel. The significant correlation found between PC1 and the latitudinal distribution of the sesame accessions—similar to findings by Wei et al. (2015)—indicates that the sesame germplasm accessions were structured on the basis of their geographic collection. According to the PC1 axis, the panel divided into two major groups, consistent with the prior structure analysis at *K* = 2. However, the PCA was unable to capture much variance in the PC1 axis, which suggests that the sesame panel contained a number of admixed lines and a low layer of population structure.

According to the AMOVA results, 14.92% of the marker variation was explained by the population structure of the sesame panel. This result suggests the absence of a complicated population structure in our association-mapping panel. The differentiation between the subgroups was further validated by the *F*_ST_ value, calculated here as 0.15, which demonstrates the moderate genetic differentiation characterizing this panel. The latter finding should favor the detection of gene effects on the power of structure-based association studies.

### Linkage Disequilibrium in Sesame

The decay of LD over a known genetic distance is an important parameter for determining the number and density of molecular markers deemed appropriate for GWAS and selection strategies (Mather et al., 2007). Sesame, as a predominantly self-pollinated species, albeit one that readily outcrosses if able, is expected to have a higher level of LD than that found in cross-pollinated crops. Wang et al. (2014) had reported that the LD decay was ∼150 kb in 29 sesame accessions. Later, an LD decay of ∼88 kb was reported in 705 sesame accessions (Wei et al., 2015). In the present study, the LD decay was estimated at ∼99 kb—the point at which the *r*^2^ dropped to 0.37 from its initial value of 0.74—and much lower than that found by Wang et al. (2014). This discrepancy may reflect the effect of a small population size, as reported by Wang et al. (2014), wherein genetic drift drives a consistent loss of rare allelic combinations, thereby increasing its LD level. Because the LD decay in our study was closer to that reported by Wei et al. (2015), it suggests that our sesame panel had an adequate number of accessions and a rich genetic diversity for GWAS.

## Conclusion

The sesame accessions used in this study were selected from the HSRC-HAAS core collection and so they mainly encompassed landraces and cultivars. All the accessions displayed good adaptation to most of the current growing conditions in China; this means that field experiments could be robustly conducted to test multiple phenotypic traits in different places throughout China. With the aim of evaluating the potential of a panel for an association analysis, we studied the population diversity and structure of a sesame panel consisting of 366 accessions. Our results show that this sesame panel is a representative sample, and one therefore suitable for further association mapping, given the modest level of nucleotide diversity and the slight population stratification that favors GWAS and a false association control.

## Author Contributions

HM and YL developed the association-mapping panel; CC and HM performed the data analysis; CC wrote the manuscript, HM revised the manuscript; HZ and YZ designed and supervised the study. All authors read and approved the final manuscript.

## Conflict of Interest Statement

The authors declare that the research was conducted in the absence of any commercial or financial relationships that could be construed as a potential conflict of interest.

## Footnotes

1 http://Biomarker.com.cn/

2 https://github.com/BGI-shenzhen/PopLDdecay
