# Corrigendum: Genetic Diversity, Population Structure, and Linkage Disequilibrium of an Association-Mapping Panel Revealed by Genome-Wide SNP Markers in Sesame

**DOI:** 10.3389/fpls.2017.02083

**Published:** 2017-11-29

**Authors:** Chengqi Cui, Hongxian Mei, Yanyang Liu, Haiyang Zhang, Yongzhan Zheng

**Affiliations:** ^1^National Key Laboratory of Crop Genetics and Germplasm Enhancement, Cotton Research Institute, Nanjing Agricultural University, Nanjing, China; ^2^Henan Sesame Research Center, Henan Academy of Agricultural Sciences, Zhengzhou, China; ^3^Key Laboratory of Oil Crops in Huanghuaihai Plain, Ministry of Agriculture, Zhengzhou, China; ^4^Henan Provincial Key Laboratory for Oil Crops Improvement, Zhengzhou, China

**Keywords:** *Sesamum indicum* L., SNPs, genetic diversity, population structure, linkage disequilibrium

In the published article, there was an error regarding the affiliation**s** for Haiyang Zhang. As well as having Affiliation 3, they should also have National Key Laboratory of Crop Genetics and Germplasm Enhancement, Cotton Research Institute, Nanjing Agricultural University, Nanjing, China. The authors apologize for this error and state that this does not change the scientific conclusions of the article in any way.

## Conflict of interest statement

The authors declare that the research was conducted in the absence of any commercial or financial relationships that could be construed as a potential conflict of interest.

