# Supplementary material for: Genetic Diversity, Population Structure, and Linkage Disequilibrium of an Association-Mapping Panel Revealed by Genome-Wide SNP Markers in Sesame
Source: Front Plant Sci. 2017 Jul 6;8:1189. doi: 10.3389/fpls.2017.01189 (PMC5498554; doi:10.3389/fpls.2017.01189)
Supplement: Supplementary file 4 [file Image_1.PDF]

Supplementary Material

Genetic diversity, population structure, and linkage disequilibrium of an association-mapping panel revealed by genome-wide SNP markers in sesame

Chengqi Cui[1†], Hongxian Mei[2†], Yanyang Liu[2], Haiyang Zhang[2*] and Yongzhan Zheng[2*]

[*] **Correspondence:**

Haiyang Zhang

haaszhy@yahoo.com;

Yongzhan Zheng

sesame168@163.com

1. Supplementary Figures

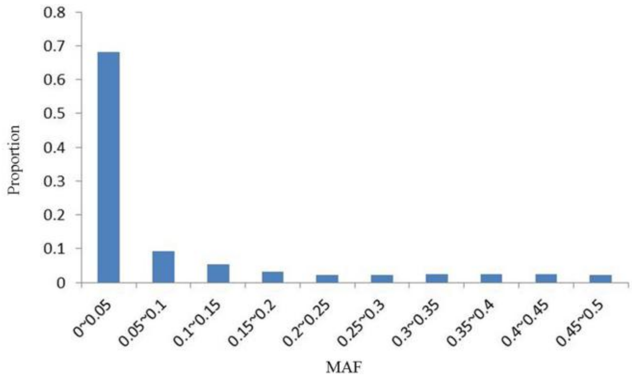

FIGURE S1 The distribution of minor allele frequencies (MAF) in the sesame panel. The *x*-axis represents the MAF, while the *y*- axis represents the proportion of MAF.

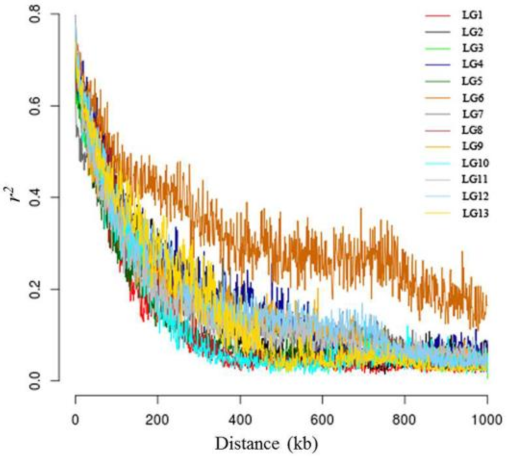

FIGURE S2 The linkage disequilibrium (LD) across the 13 linkage groups (LGs) of sesame.
